# Case Report: Hemispherotomy in the First Days of Life to Treat Drug-Resistant Lesional Epilepsy

**DOI:** 10.3389/fneur.2021.818972

**Published:** 2021-12-24

**Authors:** Konstantin L. Makridis, Christine Prager, Anna Tietze, Deniz A. Atalay, Sebastian Triller, Christian E. Elger, Ulrich-Wilhelm Thomale, Angela M. Kaindl

**Affiliations:** ^1^Department of Pediatric Neurology, Charité – Universitätsmedizin Berlin, Berlin, Germany; ^2^Center for Chronically Sick Children, Charité – Universitätsmedizin Berlin, Berlin, Germany; ^3^Institute of Cell- and Neurobiology, Charité – Universitätsmedizin Berlin, Berlin, Germany; ^4^Neuroradiology, Charité – Universitätsmedizin Berlin, Berlin, Germany; ^5^Beta Neurologie – Kompetenzzentrum für Epilepsie, Beta Klinik GmbH, Bonn, Germany; ^6^Pediatric Neurosurgery, Charité – Universitätsmedizin Berlin, Berlin, Germany

**Keywords:** hemispherotomy, epilepsy surgery, epilepsy, drug-resistant epilepsy, infant, pediatrics, EEG

## Abstract

**Background:** Neonatal drug-resistant epilepsy is often caused by perinatal epileptogenic insults such as stroke, ischemia, hemorrhage, and/or genetic defects. Rapid seizure control is particularly important for cognitive development. Since early surgical intervention and thus a short duration of epilepsy should lead to an optimal developmental outcome, we present our experience with hemispherotomy in an infant at the corrected age of 1 week.

**Methods:** We report successful hemispherotomy for drug-resistant epilepsy in an infant with hemimegalencephaly at a corrected age of 1 week.

**Results:** The infant was diagnosed with drug-resistant lesional epilepsy due to hemimegalencephaly affecting the left hemisphere. Given congruent electroclinical findings, we performed a left vertical parasagittal transventricular hemispherotomy after critical interdisciplinary discussion. No complications occurred during the surgery. Intraoperatively; 118 ml of red blood cells (30 ml/kg) and 80 ml of plasma were transfused. The patient has been seizure-free since discharge without further neurological deficits.

**Conclusion:** We demonstrate that early epilepsy surgery is a safe procedure in very young infants if performed in a specialized center experienced with age-specific surgical conditions and perioperative management. The specific surgical difficulties should be weighed against the risk of life-long developmental drawbacks of ongoing detrimental epilepsy.

## Introduction

Neonatal-onset drug-resistant epilepsy results from perinatal epileptogenic insults such as stroke, ischemia, hemorrhage, and/or genetic defects. Rapid seizure control is key for the cognitive developmental outcome of affected young individuals. Initial treatment always involves antiseizure medication (ASM), complemented sometimes later by medical diets to inhibit seizure activity. In about a third of all patients with epilepsy, however, two concomitantly or subsequently applied ASM fail to control seizure activity (1). The chance of achieving seizure freedom with additional ASM in these so-called drug-resistant patients is estimated to be as low as <5% (2). While epilepsy surgery can cure epilepsy in a subset of drug-resistant patients and contribute to a better development (3), the operative risk due to low weight, low blood volume and a different texture of the developing brain discourages most epilepsy centers from offering surgery in the 1st months of life. So far, there are few reports of infants aged 2–6 months or younger undergoing epilepsy surgery. The studies published so far are very heterogeneous in terms of type of surgery, surgical technique as well as age range (4–6). Given that an early surgical intervention and thus short duration of epilepsy should result in an optimal developmental outcome, we have focused on such epilepsy surgery cases. Here, we present our experience with hemispherotomy in an infant at a corrected age of 1 week.

## Case Report

**Figure 1:**
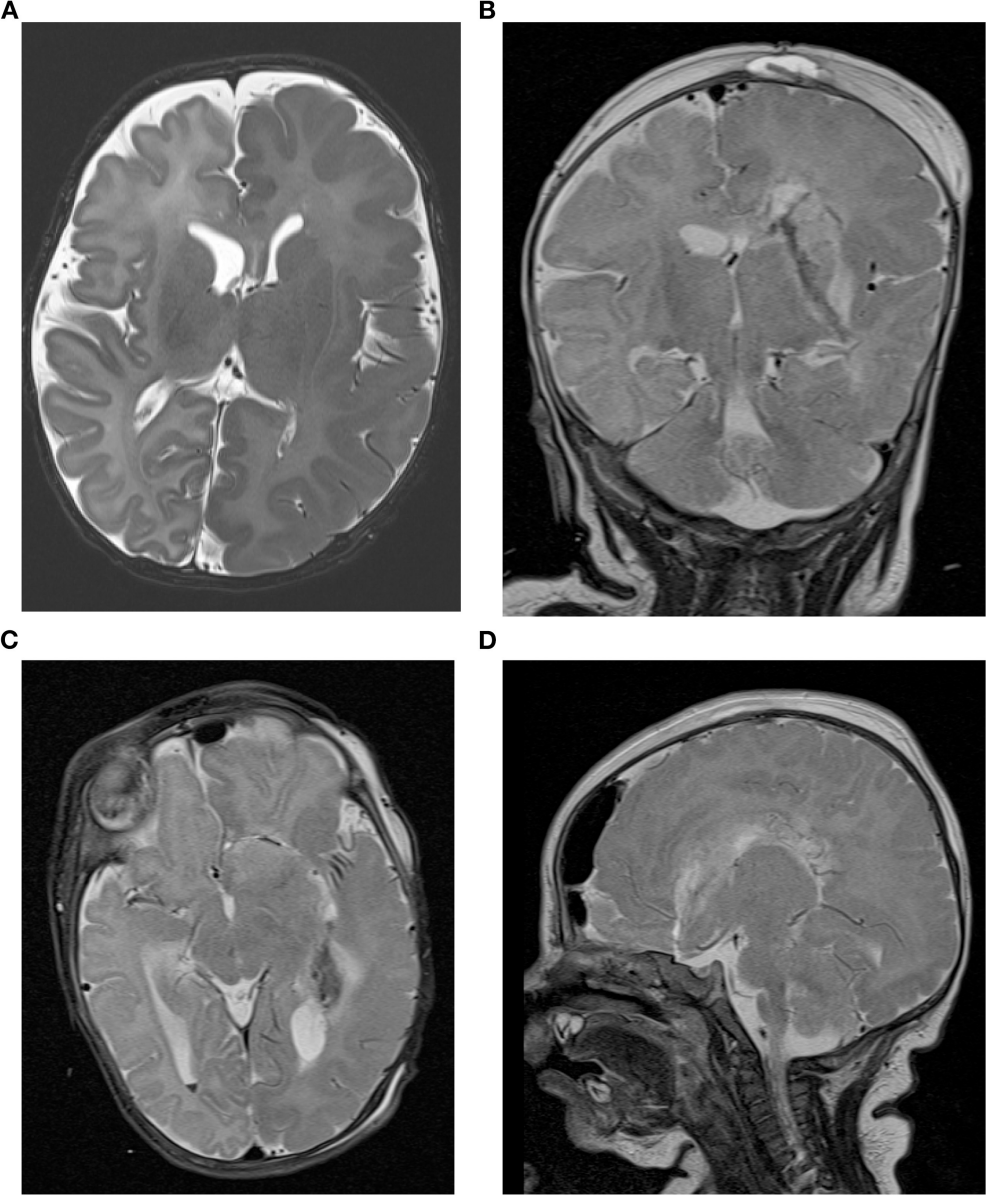
Cranial MRI of the patient. **(A)** The presurgical axial T2-weighted image shows left hemimegalencephaly with thickened cortex, an irregular gyral pattern and abnormal T2w hypointensity of the white msatter suggestive of accelerated myelination in the entire left hemisphere. Postsurgical coronal **(B)**, axial **(C)**, and sagittal **(D)** T2-weighted images demonstrate the left sided hemispherotomy.

A premature infant born at 33 weeks of gestation to non-consanguineous, healthy parents was referred to our center at the age of corrected 39 weeks (chronological age 6 weeks) for drug-resistant epilepsy. The child presented with apnea and bilateral facial clonic seizures 10 min post-partum provoking intubation and mechanical ventilation for 2 days. Focal-onset seizures affecting mainly the right-sided face and lower extremities were noted on the 7th day of life but did not respond to vitamin B6, phenobarbital, levetiracetam, and phenytoin. At the time of transfer to our intensive care unit the boy had a left-hemispheric electro-clinical status epilepticus on 55 mg/kg/d levetiracetam and 5 mg/kg/d phenobarbital, with persistent epileptic discharges over the left hemisphere with clinically visible grimacing, fasciculations, hyperextension and torsion of the upper body, and concomitant hypotension and tachycardia. An adjustment of the anticonvulsive therapy to levetiracetam (60 mg/kg/d), clobazam (0.5 mg/kg/d), lacosamide (5 mg/kg/d), and a discontinuation of phenobarbital had no sustained effect. The patient continued to have at least three seizures per day. In several VEEGs recorded over time the seizure onset could be clearly lateralized to the left hemisphere. The seizures usually start in the centroparietal and occipital region on the left side. No ictal EEG and epilepsy-typical potentials could be demarcated on the right side. Cranial MRI revealed a left-sided hemimegalencephaly with extensive gyration abnormalities, myelination abnormalities, and minor intraventricular and cortical cerebellar hemorrhage (Figure 1A). Standardized developmental assessment revealed age appropriate cognitive (DQ 105), language and fine and gross motor skills, using the Bayley Scales of Infant and Toddler Development 3rd ED.

Based on the above results, the infant was diagnosed with drug-resistant lesional epilepsy due to a hemimegalencephaly affecting the left hemisphere with congruent electroclinical findings. Following a critical discussion in our interdisciplinary pediatric epilepsy surgery board and with the parents, a left vertical parasagittal transventricular hemispherotomy (modified from Delalande technique) (7) under navigation and intraoperative ultrasound guidance was performed, at a corrected age of 1 week (chronological age 60 days) and a body weight of 3,920 g. Given the anatomical circumstances of narrow ventricles and a moderate midline shift due to the hemimegalencephaly surgery was particularly challenging to work in narrow corridors of dissecting the ipsilateral corpus callosum, fornix, insular, and anterior basal white matter tissue and not harming either central venous and middle cerebral arterial vasculatures. As standard in our epilepsy surgery program, an external ventricular drainage was not placed. However, no complications occurred during the surgical procedure that lasted 3 h and 48 min from incision to suture; 118 ml red blood cells (RBC) (30 ml/kg) and 80 ml plasma were transfused intraoperatively. No signs of mass occupying intracranial bleeding or edema were seen in a cranial MRI performed as part of the routine follow-up 24 h postoperatively (Figures 1B–D). Similarly, cranial ultrasound follow-ups performed routinely in infants post-hemispherotomy did not reveal any cerebrospinal fluid circulation problems. Postresection electrocorticography showed no epileptiform discharges on the right hemisphere. Postoperatively, the infant showed vivid bilateral movements with only a discrete right-sided muscle weakness. The patient has been seizure-free since discharge without any additional neurologic deficits at 2-month follow-up visit. The case study was approved by the Local Ethics Committee (approval no. EA2/084/18).

## Discussion

We demonstrate that epilepsy surgery in early infancy is a safe approach in very young infants if performed in a specialized center experienced with the age-specific surgical conditions and perisurgical management. The overall hemispherotomy-linked mortality has been reported to be 1.1% for the general epilepsy surgery population (8), and complications range from bleeding to meningoencephalitis to thrombosis and edema. Hydrocephalus occurs in 14% following hemisperotomy (8). Neurosurgery in infants is complicated by the immature brain texture and fragile vessels, the limited space in case of edema, the impact of blood loss considering the low total blood volume in infants and potential hemodynamic and coagulation problems due to RBC administration (9). However, only few cases have been reported and there is a lack of knowledge in this age group. Putative and largely unknown complications of an early intervention should be weighed against the risk of life-long developmental drawbacks of ongoing detrimental epilepsy and/or anticonvulsant therapy. However, early epilepsy surgery in infancy is receiving increased attention (10, 11). First results are promising with seizure freedom ranging from 66 to 82.7%. In a large proportion of patients, ASM can be discontinued. However, data on cognitive and functional outcome are lacking. Therefore, there is a need for prospective studies to determine the minimal requirements for epilepsy surgery in young infants to target and provide better care for these patients and to improve neurosurgical techniques for this special age group.

## Data Availability Statement

The raw data supporting the conclusions of this article will be made available by the authors, without undue reservation.

## Ethics Statement

The studies involving human participants were reviewed and approved by the Ethics Committee at Charité - Universitätsmedizin Berlin (approval no. EA2/084/18). Written informed consent from the participants' legal guardian/next of kin was not required to participate in this study in accordance with the national legislation and the institutional requirements.

## Author Contributions

KM and AK contributed to conception and design of the study. KM organized the database and wrote the first draft of the manuscript. All authors discussed the results, revised the first draft, and contributed to the final manuscript.

## Conflict of Interest

The authors declare that the research was conducted in the absence of any commercial or financial relationships that could be construed as a potential conflict of interest.

## Publisher's Note

All claims expressed in this article are solely those of the authors and do not necessarily represent those of their affiliated organizations, or those of the publisher, the editors and the reviewers. Any product that may be evaluated in this article, or claim that may be made by its manufacturer, is not guaranteed or endorsed by the publisher.
